# Pattern of traditional medicine use by adult Saudi patients with neurological disorders

**DOI:** 10.1186/s12906-015-0623-6

**Published:** 2015-04-01

**Authors:** Yousef Mohammad, Ahmed Al-Ahmari, Fahad Al-Dashash, Fawaz Al-Hussain, Firas Al-Masnour, Abdullah Masoud, Hoda Jradi

**Affiliations:** Department of Internal Medicine, King Saud University, Riyadh, Saudi Arabia; Department of Community Medicine, King Abdul Aziz University, Riyadh, Saudi Arabia

**Keywords:** Traditional medicine, Saudi Arabia, Neurological disorders, Cupping, Cauterization

## Abstract

**Background:**

Traditional medicine (TM) has been established as a two-edged sword. On one edge numerous forms of TM have been proven safe and effective, while on the other edge various modes of TM have been shown to be futile and potentially dangerous. Resorting to TM, especially for chronic diseases, is common world-wide and includes Saudi Arabia. Most neurological diseases are chronic. No data is available on the utilization of TM among patients with neurological disorders. We conducted this study to assess for the prevalence, pattern, perception and triggers for TM use by the adult Saudi patients with neurological disorders.

**Methods:**

A survey written in Arabic and comprised of 15 questions was used to collect data on the practice of TM among the neurology patients of King Saud University Ambulatory Clinic. The questions in the survey pertain mainly to the frequency of TM practice, its form and the patient’s opinion of this practice. The data was collected through a face to face interview by three medical students who were instructed on the survey questions prior to the launch of the study.

**Results:**

292 patients completed the survey (35.9% males and 64.0% females). 67% (n = 196) of the sample used TM. Cupping or what is commonly known as “hojamah” was the most prevalent method (45.4%) followed by herbs, skin cauterization and the Reciting of the Holy Quran (42.3%, 33.7% and 20.4% respectively). The prevalence of TM use did not differ across gender (chi-sq = 2.02; p-value = 0.15), level of education (chi-sq = 4.02; p-value = 0.40), health status (chi-sq = 2.29; p-value = 0.68), age groups (chi-sq = 5.12; p-value = 0.16), or perception toward TM (chi-sq = 2.67; p-value = 0.26) in this population.

**Conclusion:**

The practice of TM is common among the neurology patients of Saudi Arabia. Cupping, herbs, and skin cauterization, which can be harmful when wrongly employed, are frequently utilized in this patient population. Measures and policies to endorse the appropriate use of TM by Saudi society must be implemented promptly.

**Electronic supplementary material:**

The online version of this article (doi:10.1186/s12906-015-0623-6) contains supplementary material, which is available to authorized users.

## Background

The utilization of TM is highly prevalent world-wide. This practice has continued from ancient times to the present, despite the abundance of effective modern treatments. Cross-sectional studies conducted in Turkey, the United States of America, Malaysia, and Australia showed the prevalence in the use of TM of 61%, 82%, 61%, and 51% respectively [1-4]. Patients have resorted to TM in every disease entity and illness and in all forms and composition. The use of TM continues even though the efficacy of some products has never been proven, but discomfort, treatment hindrance, pain and even danger have been documented [5,6]. Some patients have used TM as the sole treatment while others combined it with the prescribed therapy. TM has been used for treatment or preventive modality [7], for acute or chronic purpose [8], for medical or psychiatric disorders, and for benign or malignant disorders [1-3,9]. The different modes of TM practiced is extensive and includes herbs, acupuncture, cupping, faith healing, homeopathy, light therapy, astrology, auto-urine therapy, massage, ear candling, hypnotherapy, yoga therapy, nutritional/vitamin therapy, natural products, and magneto therapy.

**Figure 1:**
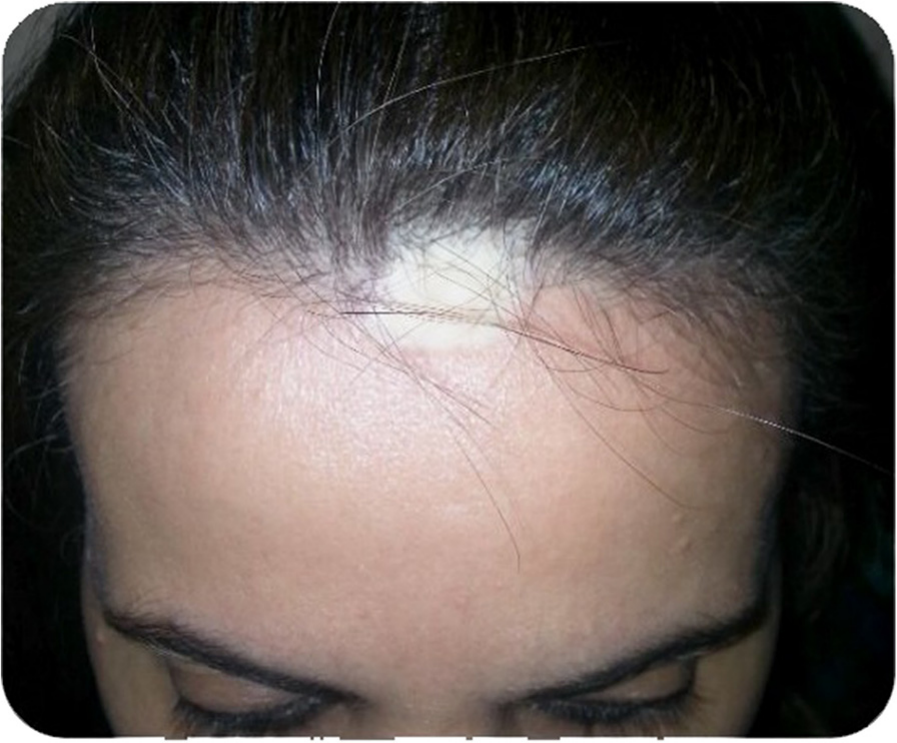
**Scalp scar of a young woman who underwent skin cauterization for epilepsy.**

**Figure 2:**
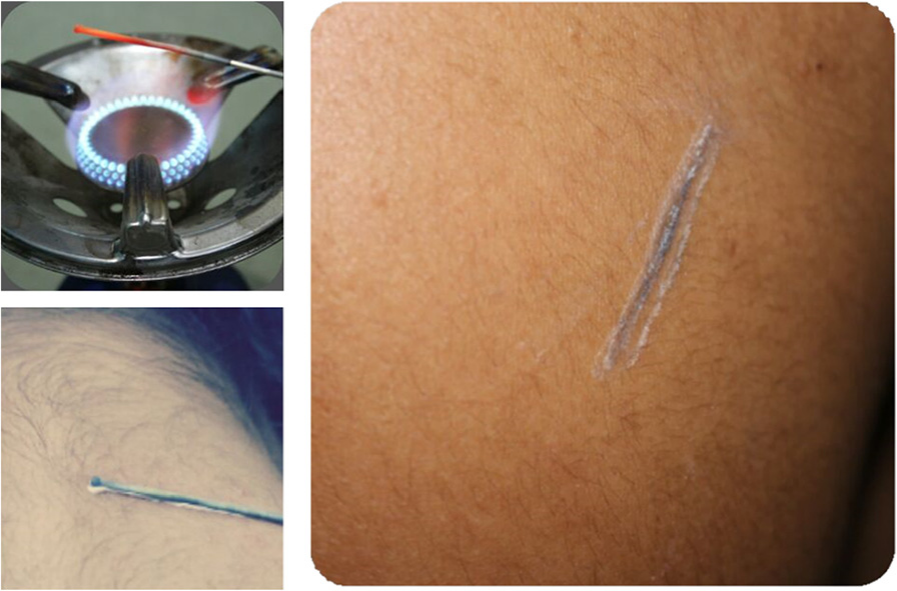
**A patient undergoing skin cauterization, by using gas stove, at the back and neck area.**

The practice of TM in Saudi Arabia, like any other country, is common and employed in every medical or psychiatric disorder. Cross-sectional studies on the use of TM in Saudi patients with cancer, liver disease, and asthma showed a prevalence of 55%, 90% and 80%, respectively [9-11]. The choice of TM in Saudi Arabia is sometimes of a disturbing, unpleasant, or even dangerous form [5,12], (Figures 1 and 2). In a study to assess the indigenous menstrual hygiene practice of Saudi girls in Riyadh, Moawed found two-thirds of the girls avoided showering, perennial care, and practiced rituals during their period [13]. Abou-Elhamd reported two cases with serious complications related to skin cauterization [5]. In another study to determine the constituents of herbal remedies and other related preparation used in Saudi Arabia, Bogusz et al. showed that 39 out of the 247 samples examined contained toxic amounts of heavy metals; including mercury and arsenic [12].

A significant proportion of neurological disorders are chronic and incurable. This would prompt patients with neurological disorders to resort to TM. In a study conducted at the out-patient pediatric clinic of a major university hospital in Saudi Arabia, Jan et al. showed that children with neurological disorders comprise a risk factor for the use of TM [8]. No data exists on the frequency of TM utilization in the adult Saudi patients with neurological disorders. We conducted this study to assess for the prevalence, pattern, perception and triggers for TM use by adult Saudi patients with neurological disorders.

## Methods

This cross-sectional study was conducted at the neurology clinic of King Khalid University Hospital, one of the largest tertiary care hospitals in Riyadh, the capital of Saudi Arabia. The study was started after the protocol was approved by the King Khalid University Hospital Institution Review Board committee. Written informed consent for participation in the study was obtained from participants prior to the collection of data. The data was collected, between February 27 and April 1, 2013, through an interview-administered questionnaire while the patients were in the waiting area of the neurology clinic. The survey — consisting of 15 questions pertaining to socio-demographic variables, frequency of TM use, pattern of use, perception of TM, and triggers for TM use — was first validated for feasibility and comprehension.

### Measures of TM Use and pattern

Patients were provided with a list of different TM modalities to choose from. The list includes skin cauterization, massage, hypnosis, naturopathy, relaxation technique, herbs, vitamins, praying, acupuncture, traditional Chinese, cupping and yoga. Respondents were also provided with a blank box to document any other mode of TM not mentioned in the list.

### Measure of Perception of TM

Patient’s perception of TM was determined through two questions: 1) in your opinion what is the worst thing that can result from the practice of TM? Patients were provided with three answers to choose from: no cure, mild complication, or death. 2) In your opinion can TM cure diseases that modern medicine cannot cure? Two answers were provided: yes or no.

### Measure of Triggers

The survey contained several questions related to the socio-demographic status. Triggers for the use of TM were determined by assessing the association between the various socio-demographic variables (age, residency location, level of education, state of health, and gender) and TM use.

### Statistical analysis

Descriptive statistics were used to assess the frequency, patterns and perceptions of TM use. Chi-square calculation was used to assess the relationship between the various socio-demographic variables and TM use. It was also employed to evaluate the relationship between perception toward TM and its use.

## Results

A total of 292 patients completed the survey. The sample was composed of 105 males (35.9%) and 187 females (64.0%). Most of the respondents (73.6%) resided in Riyadh, the capital of KSA. The majority were Saudi nationals (98.3%) and as many as 42.1% in the above 40 age group. The patients’ level of education varied from illiterate (13.7%) to holders of college degrees (30.2%). Demographic characteristics are presented in Table 1.

**Table 1:** **Demographic characteristics of study participants (N = 292)**

**Variable**	**N**	**%**
Residing in Riyadh (N = 292)	215	73.6
Saudi Nationals (N = 292)	287	98.3
Age (N = 292)		
<18	37	12.7
19-27	65	22.3
28-40	67	22.9
>40	123	42.1
Gender (N = 292)		
Males	105	35.9
Females	187	64.0
Level of education (N = 285)		
Illiterate	39	13.7
Elementary school	26	9.1
Middle school	47	16.5
Secondary school	87	30.5
College or more	86	30.2
Health status (N = 292)		
Poor	111	38.0
Not so bad	129	44.2
Excellent	52	17.8

Prevalence of use and most common methods of TM uses, as reported by respondents, are presented in Table 2. Approximately 67% (n = 196) of the respondents used TM. Many of them used it in combination with a physicians’ prescription (63.6%); while others used it as the only therapy (3.8%). Among all reported forms of TM in this population, cupping or what is commonly known “hojamah,” was the most prevalent method (45.4%), followed by herbs, skin cauterization, and reciting of the Holy Quran (42.3%, 33.7% and 20.4%, respectively). The least commonly used method of TM was acupuncture (2%). With regard to the perception assessment, 64% of the respondents think that TM can cure diseases that modern medicine is unable to and 87% believe TM is not associated with any complications.

**Table 2:** **Alternative Medicine use among study participants (N = 292)**

**Methods**	**n**	**% of all users**	**% of sample total**
Cupping (hojamah)	89	45.4	30.5
Skin ironing	69	33.7	23.6
Herbs	43	42.3	28.4
Holy Quran reciting	40	20.4	13.7
Massage	32	16.3	11.0
Vitamins and minerals	12	6.1	4.1
Acupuncture	4	2.0	1.4
Relaxation techniques	6	3.1	2.0
Total use	196	100	67.1

The prevalence of the use of TM did not differ across gender (chi-sq = 2.02; p-value = 0.15), level of education (chi-sq = 4.02; p-value = 0.40), health status (chi-sq = 2.29; p-value = 0.68), age groups (chi-sq = 5.12; p-value = 0.16), or perception toward TM (chi-sq = 2.67; p-value = 0.26) in this population.

## Discussion

This is the first study to assess the use of TM in the adult Saudi patients with neurological disorders. Our study showed that 67% of this patient population has used TM in one form or another. This is consistent with the high rate reported at the national or international level [1-4,8,9,14]. A significant proportion has resorted to cupping, herbs or skin cauterization (45.4%, 42.3%, and 33.7%, respectively), which when inappropriately utilized can be a dangerous form of TM [5,12,15]. This is much higher than what has been reported for the use in different disease entities of the same country [9-11]. This aggressive approach in the use of TM by the neurology patients is probably provoked by the chronic, disabling and incurable nature of the disease. Approximately 64% of the patients think that TM can cure diseases that modern medicine cannot. This positive perception of TM is similar to what has been reported [6]. None of the socio-demographic or the perception variable was associated with TM use. This is different from what has been reported for the use in different disease entities. For example, Thomson et al. in a cross-sectional study showed that being female and being younger is a predictor for TM use [4]. In another study done in Malaysia, Saibul et al. showed that years of education were associated with TM use. Other studies conducted in Saudi Arabia showed that the risk for TM use includes age greater than 60, being a female, illiterate, young age, dissatisfaction with physician diagnosis, failure of medical treatment, long waiting time for physicians, and chronic medical conditions [8,11,14,16]. The chronic, disabling and incurable nature of the neurological disorder is the likely reason for the desperate behavior of the patients for resorting to TM, irrespective of gender, age, residence and level of education.

In our study, patients were recruited from King Khalid Specialty Hospital in Riyadh, the capital of the country and a large metropolitan city. A significant proportion of the respondents (25%) come from rural areas across the country. This is highly indicative that the sample is representative of the patient population.

A major limitation for the study relates to the measures we used to assess for the triggers of TM use and the perception of TM. These measures are insufficient and do not capture all the constructs of these two variables. The purpose of the study is to assess the pattern of TM use in this patient population. We believe this was properly determined.

The results of our study showed that Saudi patients with neurological disorders commonly utilize TM; especially the potentially unsafe forms (cupping, herbs and skin cauterization). It also revealed a diffuse misconception on the efficacy and safety of this option. Hence, extra measures must be implemented to educate the public on the safe and effective use of TM, especially those related to cupping, herbs and skin cauterization. Efforts must also be implemented to educate the primary care physicians; as many of the primary care physicians are not familiar with the majority of TM modalities, don’t feel comfortable counseling patients about many of the TM used, and even >50% of them have been utilizing TM on themselves or their families [17]. Moreover, as per the recent WHO recommendations, the public health agency of the country must develop policies that will promote and ensure the proper use of TM practices [18].

## Conclusion

Our study showed that the practice of traditional medicine is common among the adult Saudi patients with neurological disorders. Alarmingly, the potentially dangerous forms of traditional medicine (cupping, herbs and cauterization) are the most frequently utilized by this patient population. Measures and policies to endorse the appropriate use of TM by the Saudi society must be implemented promptly.

## Consent

Formal consent to publish the patient images in the manuscript was obtained from the patients featured.
